# Role of Lactate in Inflammatory Processes: Friend or Foe

**DOI:** 10.3389/fimmu.2021.808799

**Published:** 2022-01-14

**Authors:** Carolina Manosalva, John Quiroga, Alejandra I. Hidalgo, Pablo Alarcón, Nicolás Ansoleaga, María Angélica Hidalgo, Rafael Agustín Burgos

**Affiliations:** ^1^ Faculty of Sciences, Institute of Pharmacy, Universidad Austral de Chile, Valdivia, Chile; ^2^ Laboratory of Immunometabolism, Faculty of Veterinary Sciences, Institute of Pharmacology and Morphophysiology, Universidad Austral de Chile, Valdivia, Chile; ^3^ Graduate School, Faculty of Veterinary Sciences, Universidad Austral de Chile, Valdivia, Chile

**Keywords:** lactate, inflammation, G-protein coupled receptors, immunometabolism, monocarboxylate transport

## Abstract

During an inflammatory process, shift in the cellular metabolism associated with an increase in extracellular acidification are well-known features. This pH drop in the inflamed tissue is largely attributed to the presence of lactate by an increase in glycolysis. In recent years, evidence has accumulated describing the role of lactate in inflammatory processes; however, there are differences as to whether lactate can currently be considered a pro- or anti-inflammatory mediator. Herein, we review these recent advances on the pleiotropic effects of lactate on the inflammatory process. Taken together, the evidence suggests that lactate could exert differential effects depending on the metabolic status, cell type in which the effects of lactate are studied, and the pathological process analyzed. Additionally, various targets, including post-translational modifications, G-protein coupled receptor and transcription factor activation such as NF-κB and HIF-1, allow lactate to modulate signaling pathways that control the expression of cytokines, chemokines, adhesion molecules, and several enzymes associated with immune response and metabolism. Altogether, this would explain its varied effects on inflammatory processes beyond its well-known role as a waste product of metabolism.

## Introduction

Lactate is a hydroxycarboxylic acid that is present as two stereoisomers in mammals, the left-handed (L-lactate) and the right-handed (D-lactate) forms, with L-lactate being the predominant form produced during anaerobic glycolysis (1, 2). Several lines of evidence suggest that activation of inflammatory immune cells induces a shift from oxidative phosphorylation towards aerobic glycolysis with an increase in lactate, like the Warburg effect observed in tumor cells. The increase in L-lactate production is a metabolic response observed in activated neutrophils, macrophages, and dendritic cells using Toll-like receptors (TLR) ligands or pro-inflammatory cytokines (3). Additionally, the presence of lactate has been proposed as a biomarker in various diseases, including neoplasia malignancy, sepsis, and autoimmune diseases (4–6).

Conversely, D-lactate is formed through the methylglyoxal pathway in nanomolar concentrations (7) but increase under pathophysiological conditions in humans, such as short-bowel syndrome (8, 9), fatigue syndrome (10), diabetes mellitus (11), propylene glycol intoxication (12) and in patients harboring deleterious enzymatic variants producing poor metabolizers of D-lactate (13). In cattle, several pathologies are associated with an increase in D-lactate during acute ruminal acidosis (ARA). ARA is produced by an imbalance of ruminal bacteria after consuming excessive, highly fermentable carbohydrates. This ingestion of carbohydrates is followed by the proliferation of lactate-producing microorganisms, such as *Streptococcus bovis*, which metabolizes carbohydrates to L (+) and D (-) lactate. Bovines with ARA develop several lesions, including ruminitis, polioencephalomalacia (calves), liver abscess, and lameness (14). The evidence suggest that lactate would be involved in the pathophysiology of lameness in cattle (14). In fact, D-lactic acidosis has been closely associated with the appearance of laminitis and polysynovitis (15–19).

Lactate is often considered a metabolic waste of glycolysis; however, several studies suggest that lactate, is more than an intermediate metabolite, exerting immunomodulatory pleiotropic effects that regulate the inflammatory response. The aim of the review is to provide evidence on the various roles of lactate in inflammatory processes, which would depend on the metabolic status, the cellular phenotype, as well as the presence of a myriad of receptors that could modulate its effects.

## Sources, Metabolism, and Transport of l-Lactate and d-Lactate in Mammals

### Sources and Metabolism

During glycolysis, glucose is metabolized into two pyruvate molecules with the associated production of two ATP and two NADH molecules (20). Under the presence of oxygen, pyruvate is converted to acetyl-CoA by pyruvate dehydrogenase (PDH) in the tricarboxylic acid (TCA) cycle, producing approximately 25 ATP molecules per molecule of glucose (20). Under oxygen-deprived conditions, by the fermentative branch of the glycolytic pathway (21), NADH is used to reduce pyruvate to lactate through cytosolic lactate dehydrogenase (LDH), a process that results in two ATP molecules and two lactate molecules without consuming oxygen (20). However, this last conversion of pyruvate to lactate also occurs under aerobic conditions and not only under conditions of lack of oxygen, which is known as the ‘Warburg effect’ (21).

**Figure 1:**
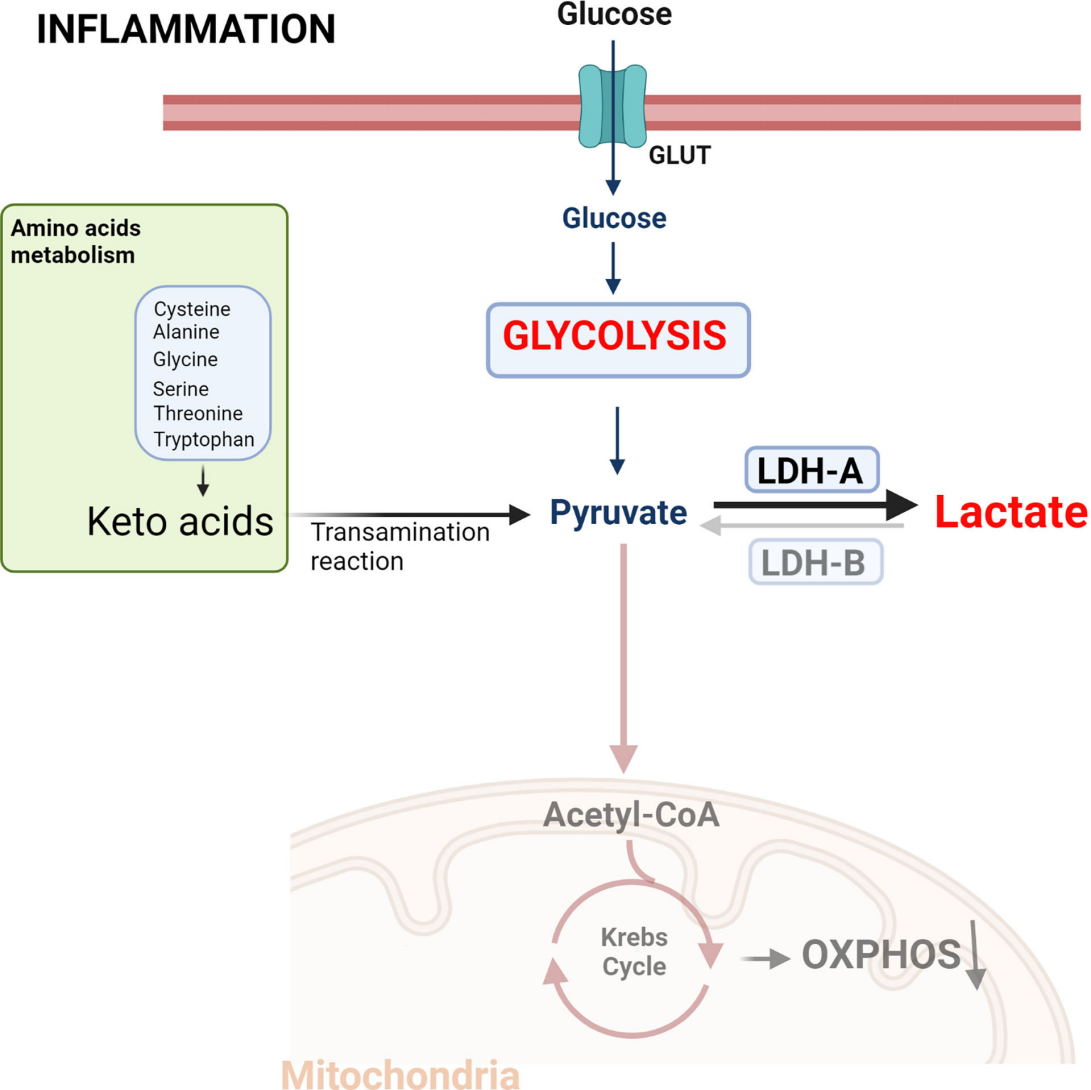
Metabolic reprogramming induced by inflammatory signals. The increased energy required by activated immune cells leads to a metabolic change characterized by aerobic glycolysis, increased lactate production and a reduction in the use of the tricarboxylic acid (TCA) cycle. This metabolic change is equivalent to the phenotype exhibited by cancer cells defined as the Warburg effect. Created with BioRender.com.

Pro-inflammatory signals induce cellular metabolic changes, characterized by increased glycolysis in the presence of oxygen, in a similar fashion to Warburg effect. In this way, the pyruvate formed during this process is reduced by LDH-A to lactate at the expense of a disruption of the TCA cycle and oxidative phosphorylation (OXPHOS) **(** **Figure 1** **).** Overall, metabolic reprogramming is essential for both the inflammatory and anti-inflammatory response in immune cells, with aerobic glycolysis being predominant in inflammatory processes, while OXPHOS is more related to the anti-inflammatory response (22–25).

LDH is a nicotinamide adenine dinucleotide (NAD+) dependent enzyme that mediates the bidirectional conversion of pyruvate and lactate concomitantly with the oxidation and reduction of the cofactor NAD+. LDH is a tetramer made up of two subunits, LDH-A and LDH-B, LDH-A having higher affinity and Vmax for pyruvate than LDH-B. Thus, LDH-A, and particularly the LDH-5 tetramer (consisting of 4 subunits of LDH-A), catabolizes pyruvate to lactate. In contrast, LDH-B transform lactate into pyruvate, allowing cells to use lactate as a source of nutrients for oxidative metabolism (26). Both L- and D-stereoisomers of lactate are produced and metabolized to pyruvate through the enzyme LDH. However, LDH has stereoselectivity, so D-lactate production and metabolism require D-LDH, and L-lactate requires L-LDH (20, 27, 28). The physiological serum concentration of lactate is 1 to 2 mM, with L-lactate being the predominant physiological enantiomer. Plasma ratio of D-lactate versus L-lactate is estimated to be 1: 100 under normal conditions (29). L-LDH catalyzes a bidirectional reaction between L-lactate and pyruvate, however, human D-LDH catalyzes the one-way conversion from D-lactate to pyruvate (29). L-lactate is produced by L-LDH-mediated oxidation of pyruvate obtained from glucose (65%) and alanine (16–20%) metabolism. To a lesser extent, pyruvate can also be produced during serine, threonine, and cysteine catabolism **(** **Figure 1** **)** (30). In contrast, D-LDH does not catalyze the conversion of pyruvate to D-lactate and other sources give rise to this enantiomer. L-lactate is rapidly metabolized to pyruvate in the cytosol and within the mitochondria by L-LDH, while the metabolism of D-lactate takes place only in the inner side of the mitochondria by D-LDH (24, 25) giving reduction equivalents to complex III of the respiratory chain (31).

An increase of LDH-A expression is key in the metabolism of cancer cells and is considered a negative prognostic biomarker (32, 33). LDH-A is upregulated in CD8+ T cells and fibroblast-like synoviocytes (FLSs) from rheumatoid arthritis patient and is considered a potential target to rewire the metabolism and reduces inflammatory response (34–37). Recently, D-LDH has been linked to pathological conditions. Differential tissue expression of D-LDH could be involved in some neuropathological conditions (13). D-lactic acidosis in calves produces several neurological signs, such as changes in behavior and posture, progressing to coma and recumbency correlated with serum D-lactate concentrations, however, it is unknown whether this increase in D-lactate is related to defective D-LDH (38, 39). D-lactate encephalopathy is a rare reversible neurologic syndrome that occurs in individuals with short bowel syndrome (40–42). D-lactate inhibits mitochondrial respiration in the brain, possibly due to low D-LDH activity, which interferes with the use of pyruvate and L-lactate as substrates of mitochondrial respiration (43). D-lactate also can exert as an astrocytic metabolic inhibitor and contribute with the D-lactate encephalopathy (44). Also, in patients harboring a mutations in D-LDH that results in increased blood levels of D-lactate, it has been associated with mild cerebellar ataxia, hypotonia, cognitive impairment (45), hyperuricemia and gout (43, 46).

**Figure 2:**
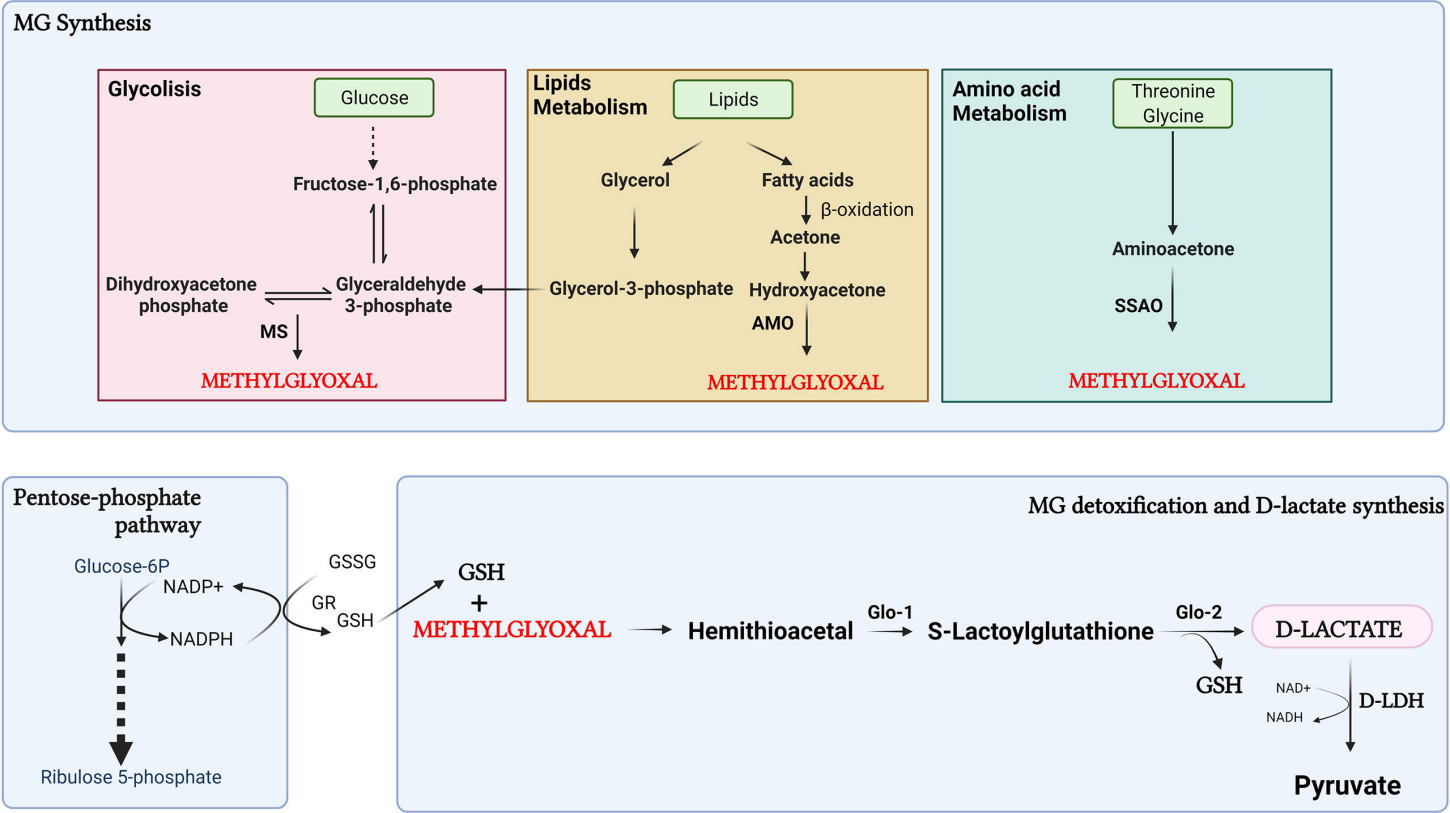
Synthesis of D-lactate through the methylglyoxal pathway. D-lactate is derived from carbohydrate metabolism through the formation of methylglyoxal (MG). Furthermore, MG can be derived from protein catabolism with formation of aminoacetone, and lipids metabolism, through reactions catalyzed by glycerol kinase and glycerol-3-phosphate dehydrogenase. In the process of detoxification of MG, D-lactate is produced. MS, methylglyoxal synthase; AMO, acetyl monooxygenase; SSAO, semicarbazide-sensitive amine oxidase; GR, glutathione reductase; GSSG, oxidized form of glutathione; GSH, reduced glutathione Glo1, Glyoxalase-1; Glo2, Glyoxalase-2. Created with BioRender.com.

D-lactate is derived from carbohydrate metabolism and lipids through the formation of methylglyoxal (MG) (47) **(** **Figure 2** **)**. MG is a by-product of glycolysis, produced by the fragmentation of dihydroxyacetone phosphate (DHAP), and glyceraldehyde 3-phosphate (G3P) (48). G3P is metabolized by triosephosphate isomerase to DHAP, whereas methylglyoxal synthase (MS) catalyzes the conversion of DHAP to MG (49). Furthermore, MG can be derived from protein metabolism, through aminoacetone formation, and from lipids, through reactions catalyzed in the kidney and liver by glycerol kinase and glycerol-3-phosphate dehydrogenase connecting glycolysis with lipid metabolism (50).

The production of MG from the oxidation of fatty acids occurs by conversion of acetone to MG in two steps catalyzed by acetone and acetyl monooxygenase (AMO). MG can also be produced by semicarbazide-sensitive amine oxidase (SSAO)-catalyzed aminoacetone deamination from the catabolism of L-threonine and glycine (49). Glycerol has been shown to be a source of D-lactate derived from MG metabolism more efficient than glucose (51). This suggests that lipolysis could be a significant additional source of MG in the cell. Also, MG can be derived from lipid peroxidation or body ketone oxidation under pathological conditions such as diabetic ketoacidosis, prolonged fasting, or a low-carbohydrate diet (48, 52). In the process of detoxification of MG, D-lactate is produced. MG is detoxified by glyoxalase-1 (Glo-1) and glyoxalase-2 (Glo-2). The first step consists of the spontaneous reaction between MG and reduced glutathione (GSH) to form a hemithioacetal, which is the substrate for Glo-1 to form S-lactoylglutathione. Glo-2 then transform S-lactoylglutathione to D-lactate, restoring GSH during this process (53). For GSH recycling, participation of the pentose phosphate pathway is important for the formation of NADPH to reduce oxidized glutathione (GSSG) through the action of glutathione reductase (GR) (53). In dairy cows with ketoacidosis, MG participates in the inflammatory response associated with this metabolic disturbance. Despite this, the presence of D-lactate in cattle with ketoacidosis has not yet been reported, and the effect of MG can not be related to the formation of D-lactate (54).

Another source of lactate L and D in mammals is its production by intestinal bacteria. In humans, strains of lactic acid bacteria (LAB) exert health-promoting functions such as immunomodulatory improvement of intestinal integrity, resistance to pathogens, prevention of lactose intolerance, anticancer effects, reduction of depression and anxiety symptoms, anti-obesity and anti-diabetic activities, and decrease serum cholesterol levels (55). L-lactate in drinking water can suppress colitis induced by dextran sulfate sodium in mice, promoting epithelial cell migration and repair through the augment of mitochondrial ATP production (56). On the other hand, microbiota-derived lactate, DL-lactate, regulates gut epithelium development, whereas LAB lacking LDH fails to induce intestinal stem-cell regeneration (57). Conversely, an increase in the abundance of some LAB strain, including *Streptococcus*, *Lactobacillus*, and *Lactococcus* has been observed in gastric cancer patients. This could involve the supply of exogenous lactate, which is an energy source for cancer cells that favor inflammation, angiogenesis, metastasis, epithelial-mesenchymal transition, and immune evasion (58). LABs are inducers of reactive oxygen species (ROS) production in cultured cells and *in vivo* (59), inducing DNA damage in colon cells (60), which contrasts with LAB effects on the gastrointestinal tract. These ambivalent effects could be attributable to the LAB strain, rather than to the effects of lactate itself (60).

In cattle, L-lactate and D-lactate are produced in the rumen by *Streptococcus bovis* and lactobacillus bacteria and are degraded by lactate-utilizing bacteria in the rumen, such as *Megasphaera elsdenii* and *Selenomonas ruminantium*, with the ruminal pH level being key in the control of the net balance of either stereoisomer (61).

### Lactate Transport

Lactate can cross the cell membrane by three known pathways: 1) free diffusion of undissociated acid, 2) exchange for another anion, and 3) transport *via* a stereospecific pH-sensitive transport protein (monocarboxylate transporter). For monocarboxylate transporters, members of the 16A solute carrier family proton-bound monocarboxylic acid symporters, i.e., MCT1 (SLC16A1), MCT2 (SLC16A7), MCT3 (SLC16A8), and MCT4 (SLC16A3) and two sodium-coupled lactate cotransporters (SLC5A12, SLC5A8) have been described (62, 63).

In humans, MCTs have been reported in retina, muscle, kidney, brain capillary endothelial cells, cardiac myocytes, enterocytes, hepatocytes, erythrocytes, thymocytes, placenta, and nervous tissue (64, 65). In cattle, some MCT isoforms have been described in the rumen epithelium (66), neutrophils (67), and fibroblast-like synoviocytes (bFLS) (17). In mammals, MCT1 is ubiquitous and take part in lactate uptake in neutrophils (67) and in various organs such as the heart, skeletal muscle, and red blood cells, as well as in the liver for gluconeogenesis (68). MCT2 is less ubiquitous and plays an important role in neurons, and MCT3 has been identified only in the retinal pigment epithelium and the choroid plexus epithelium (68). Conversely, MCT4 is expressed in strongly glycolytic cells, such as muscle fibers, and has been shown to increase its expression in response to hypoxia (69). In human immune cells, MCT1, MCT2, and MCT4, in granulocytes, lymphocytes, and monocytes have been detected (70). Similarly, in bovine neutrophils, both MCT1 and MCT4 mRNA and proteins are expressed; however, MCT2 and MCT3 are absent (67). MCT4 expression levels in the bovine neutrophil were 1,000 times lower than MCT1 levels (67). MCT1 and MCT4 are associated with the chaperone CD147, which organizes both the distribution and the location of both transporters in the membrane (71, 72). In fact, the presence of the chaperone protein CD147 has also been detected in bovine neutrophils (67).

MCT1 plays an essential role in neuroinflammation since lipopolysaccharide (LPS) was shown to increase the expression of MCT1 and 6-phosphofructo-2-kinase/fructose-2,6-biphosphatase 3 in microglia obtained from the brain of C57BL/6 mouse (73). The knockdown of MCT1 suppressed the glycolysis rate and decreased the LPS-induced expression of inducible nitric oxide synthase (iNOS), interleukin (IL)-1β, IL-6, and STAT1 phosphorylation in BV2 microglial cells (73). MCT4 is up-regulated in FLS obtained from patients with rheumatoid arthritis (RA) and exports intracellular lactate into synovial fluid in the joint (74). TLR2 and TLR4 agonists up-regulate MCT4 in human and mouse macrophages (75). Increased expression of MCT4 is mediated by MYD88 in an NF-κB-dependent manner and is necessary for the sustained high glycolysis observed during macrophage activation (75).

The transport kinetics for both stereoisomers have been measured in frog oocytes expressing MCT1 and MCT4. The Km values for MCT1 determined for L-lactate and D-lactate were 4.4 and >60 mM and for MCT4 were 28 and 519 mM, respectively (76). Despite the different Km of both stereoisomers D-lactate has pro-inflammatory effects dependent on MCT1 in both neutrophils and bFLS, which suggests that the uptake of D-lactate by MCT1 is a requirement for the pro-inflammatory effects of this stereoisomer (17, 67). Accordingly, bovine with acute ruminal acidosis (ARA) 5 mM of D-lactate versus 1.6 mM of L-lactate in the bloodstream has been determined (77). It has been suggested that D-lactate is rapidly absorbed but metabolized more slowly than L-lactate by bovine tissues (78). Therefore, only D-lactate blood concentrations are increased in cattle with ARA (77, 79), which could contribute to the development of inflammatory processes in ruminants.

## Lactate as a Cellular Metabolite Immunomodulator

To carry out the inflammatory response, immune cells must activate metabolic pathways as part of host defense responses (80). Conversely, each population of immune cells requires different metabolism and nutrient use (81). It has been shown that macrophage metabolism can influence inflammatory cytokine production, and the same has been demonstrated in T cells (82), myeloid-derived suppressor cells (MDSC) (83), and dendritic cells (DC) (84). The intersection between metabolism and immunity has been proposed to be part of the field of immunometabolism (81). Most research has focused on the absorption and metabolism of glucose, amino acids, mainly glutamine, and certain fatty acids. However, lactate, the end product of the glycolytic pathway, may regulate the inflammatory response in various cells (85). Lactate is produced and secreted in significant quantities by immune cells during the inflammatory process (3, 85). Although short-term lactate exposure has limited effects on cytokine production, long-term lactate treatment shows strong anti-inflammatory effects in monocytes (85). This adaptation of immune cells to lactate concentrations in the microenvironment, may affect the functions of tissue-specific immune cells (85). Moreover, it suggests that the duration of lactate exposure can also decide the outcome of immunomodulatory effects.

It has recently been demonstrated in neutrophils that lactate can be used as non-glucose substrates to generate glycogen stores (86). In addition, in neutrophils, LPS increases the gluconeogenesis that fuels glycogen deposition, which in turn support the higher energy demands of a pro-inflammatory response (86). In FLS, a relevant effector of synovial immunity (87), an increase in lactate production is associated with the characteristic glycolytic metabolic rewiring in rheumatoid arthritis (88); moreover, lactate can induce metabolic reprograming in FLS and increase pro-inflammatory cytokine expression (89). Altogether, these results suggest that the effect of lactate on inflammation is closely related to the cellular phenotype and its metabolic status.

### Lactate Activity in Endothelial Cells

Endothelial cells (ECs) control the extravasation of circulating immune cells into tissues through the production of cytokines, chemokines, and adhesion molecules. It has been proposed that lactate can activate signaling pathways in endothelial cells and modulate the inflammatory response.

**Figure 3:**
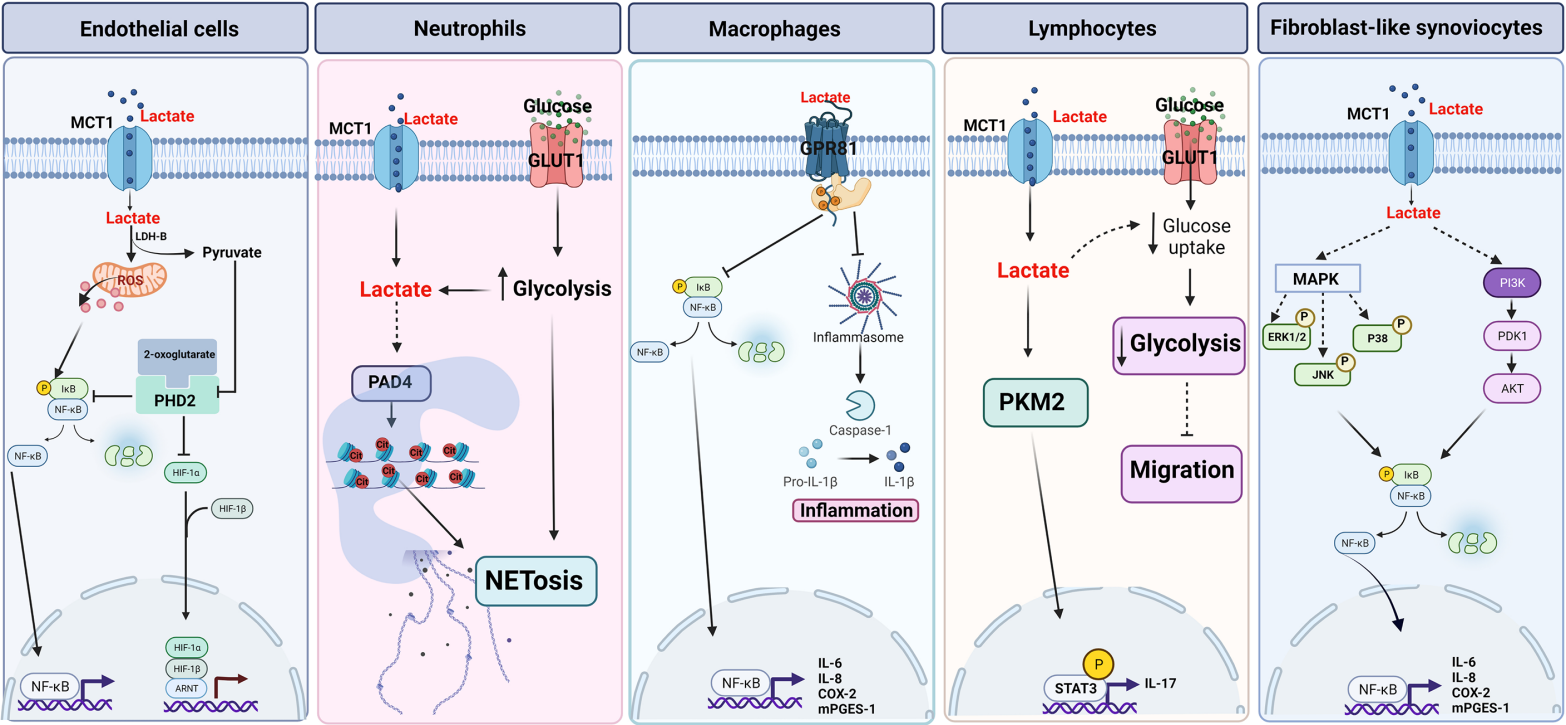
Proposed lactate signaling pathways. ***Endothelial cells***. Lactate activates NF-κB pathway through the formation of reactive oxygen species (ROS). Lactate-induced HIF-1α stability is dependent on prolyl-hydroxylase (PHD). ***Neutrophils***. Lactate induces NETosis through MCT1-dependent PAD4 activation and glycolysis. ***Macrophages*.** Lactate has an anti-inflammatory effect through the activation of the GPR81 receptor and the inhibition of the inflammasome and the NF-κB pathway. ***Lymphocytes***. Lactate induces IL-17 expression in a MCT1-dependent manner and decreases migration through indirect inhibition of glycolysis. ***FLS***. Lactate regulates the expression of pro-inflammatory cytokines through MAPK and NF-κB pathways dependent on lactate input by MCT1. Created with BioRender.com.

In ECs, lactate influx *via* MCT1 induces the activation of NF-κB (90). NF-κB is constituted by p65 (RelA) and p50 (NF-κB1) subunits and remains inactive in the cytosol forming a complex with IκB proteins. Phosphorylation of IκB is crucial for the polyubiquitination and proteasomal degradation of IκB and activation of NF-κB (91). Lactate through phosphorylation and degradation of IκBα activates NF-κB and regulates a wide variety of inflammatory genes including IL-8. The activation of NF-κB by lactate is dependent on the inhibition of prolyl hydroxylase (PHD), which are Fe (II) and 2-oxoglutarate-dependent dioxygenases. The oxidation of lactate by LDH-B increases the intracellular pool of pyruvate that competes with 2-oxoglutarate and inhibits the hydroxylase activity of PHD. PDH-catalyzed hydroxylation of proline induces polyubiquitylation and degradation of HIF-1α in the proteasome. Inhibition of PDH by pyruvate results in protein stabilization of HIF-1α allowing migration to the nucleus to modulate transcription of target genes in ECs (90). These genes include proangiogenic effectors such as vascular endothelial growth factor (VEGF) (92). Overall, in ECs lactate activates both NF-κB and HIF-1α and the decrease in the catalytic activity of PHD is required for the activation of both transcription factors by lactate **(** **Figure 3** **).** However, lactate-induced NF-κB activity was also inhibited by antioxidant agents, suggesting the participation of reactive oxygen species (ROS) in the regulation of the lactate-induced inflammatory response in ECs (90). On the other hand, endothelial cells express the G-protein-coupled receptor (GPCR), GPR4, a proton-sensing receptor. Extracellular acidification by lactic acid can promotes the pro-inflammatory response in ECs, the cellular mechanisms associated with this receptor are discussed in section 4.

### Lactate Activity in Neutrophils

Neutrophils are one the first leukocytes to be recruited to the site of infection and to execute microbial killing perform diverse functions such as phagocytosis, oxidative burst (ROS) and neutrophil extracellular traps (NETs) (93). However, also neutrophil responses lead to tissue injury and are associated with several diseases such as sepsis, asthma, ischemia-reperfusion injury, and rheumatoid arthritis (94). Due to the low abundance of mitochondria, it has been commonly considered that neutrophils only use glycolysis for their biological functions (95). However, different metabolic routes are required to fulfill the energetic, biosynthetic, and functional requirements of neutrophils, including the TCA cycle, oxidative phosphorylation (OXPHOS), the pentose phosphate pathway (PPP), and fatty acid oxidation (FAO) (96, 97). Nonetheless, glycolysis is the main metabolic pathway involved in phagocytosis, ROS, and NET release (98–100). Inducers of ROS-dependent and ROS-independent NETs cause an increase in extracellular acidification rate (ECAR), LDH activity, and a reduction in pyruvate kinase M2 (PKM2) activity, increasing lactate formation (101). The deamination of histones (mainly conversion of arginine side chains to citrullines) is a key step to allow whole NET dispersion (102). In fact, mice lacking peptidylarginine deiminase 4 (PAD4), the enzyme required for histone deamination, have decreased NETosis (103). Furthermore, D-lactate induces the release of NET in bovine neutrophils, activating PAD4 and by a mechanism independent of ROS **(** **Figure 3** **)** (67).

Inhibition of lactate formation by sodium oxamate, an LDH inhibitor, reduces tachyzoite-induced NETs (104). Furthermore, sodium oxamate, inhibits NETosis and lactate accumulation during LPS-induced sepsis in mice, suggesting the importance of lactate as a pro-inflammatory agent in an experimental model of NETosis (101). In fact, both L- and D-lactate can directly induce the release of NETs (67, 101). D-lactate-induced NETosis is dependent on MCT1, and this effect favors the adhesion of PMN to the endothelium, suggesting that lactate could behave as an intracellular signaling agent (67).

D-lactate has been shown to have a pro-inflammatory effect on bovine neutrophils since it induces the release of MMP-9, increases the expression of CD11b and decreases the expression of L-selectin, which favors endothelial adhesion (67, 105). Conversely, exogenous lactate treatment also induces NET formation in human neutrophils, while inhibition of LDH activity significantly reduces NETosis (101).

### Lactate Activity in Macrophages and Mast Cells

LPS induces differentiation to M1-type macrophages, a proinflammatory phenotype that generates ATP and lactate release through aerobic glycolysis (106). Furthermore, inhibition of LDH-A by FX11 reduces lactate secretion, pro-inflammatory cytokine release, iNOS levels, and COX2 expression in RAW264.7 macrophages treated with LPS (35). Additionally, MCT1 is expressed in macrophages and increases with inflammatory stimuli such as LPS, tumor necrosis factor (TNF)-α, or nitric oxide (NO), increasing lactate uptake (107).

Macrophages exposed to 20 mM lactate for 24 h, followed by LPS plus lactate for another 24 h, increases pro-inflammatory cytokine production through MD-2 up-regulation, a TLR4 co-receptor being this response MCT dependent (108). The inhibition of MCTs by alpha cyano-4-hydroxycinnamic acid decreases the expression of lactate-induced pro-inflammatory cytokines in human macrophages, indicating that lactate transport through MCT is necessary for lactate effects on macrophages (26). Taken together, the current background suggests that lactate may be a metabolite involved in the regulation of the pro-inflammatory response in macrophages. Sodium lactate increases the LPS-induced expression of MMP-1, IL-1β, and IL-6 in U937 macrophage-like cells through NF-κB and Mitogen-activated protein kinases (MAPK) cascades (109). Other author suggest that lactic acid reduces the activity of NF-κB and the expression of pro-inflammatory cytokines presenting anti-inflammatory effects in LPS-stimulated macrophages through of GPR81 (110, 111). Moreover, an increase of protons could also explain the discrepancy of lactate effect on macrophages. Has been demonstrated that the neutralization of pH in the lactic acid-containing medium increases LPS-induced MMP-1 secretion, indicating that lactic acid-induced pH reduction may interfere with pro-inflammatory effects (109). Besides, secretion of TNF-α by macrophages is inhibited by acidic pH (112). Additionally, long term effect of exogenous lactate in M1 macrophages, trigger an endogenous ‘lactate clock’ that induce an M2 phenotype by metabolic reprograming through the epigenetic mechanism by lactylation and acetylation of histone H3 (113, 114). This effect could to assist with repairing collateral damage produced by the host during infection and to explain the anti-inflammatory effects of lactate after 24 h (85, 115, 116). Also, lactate through ERK-STAT3 signaling pathway (117) and the combination of lactate and hypoxia *via* HIF1α stabilization (118) shift the polarization into M2-like macrophages.

Similarly, lactate decreases the expression of cytokines induced by LPS and IL-33 in mouse mast cells (119, 120). The anti-inflammatory effects presented by lactate in mast cells are through a decrease in the phosphorylation of TGF-β-activated kinase-1, JNK and ERK which suppresses the activation of NF-κB (119, 120). Furthermore, the effects of lactate have been related to HIF-1α, which regulates microRNA miR-155, considered a pro-inflammatory agent in various systems (120). Other mechanisms have also been proposed, such as the reduction of the IgE-induced phosphorylation of Syk, Btk and ERK, which are signals of inflammatory responses, which confirms the anti-inflammatory role of lactate in mast cells (121).

### Lactate Activity in Lymphocytes

Several studies have shown that activated T lymphocytes increase intracellular lactate concentrations coupled with increased expression of glucose and lactate transporters (122, 123), glucose uptake (124), expression of glycolytic enzymes, and LDH (125). The foregoing has led to the conclusion that the energy required by active T cells leads to a metabolic shift toward aerobic glycolysis and an increase in lactate (126). Increased intracellular lactate production correlates with increased extracellular lactate (127), which can inhibit glucose consumption by reversing lactate flux and, in this way, interfere with T-cell function (128). Overall, the extracellular lactate uptake, through MCT, down-regulates hexokinase 1 (Hk1) which reduces glycolysis and reduces T cell migration (129). Another lactate-induced effect on T cells is increased production of the pro-inflammatory cytokine, IL-17. Both decreased migration and increased production of IL-17 induced by lactate in T cells is the hallmark of T cell – mediated inflammation in chronic inflammatory diseases (CID) (129). Consistent with the above, chronically inflamed synovial tissue from RA patients is associated with high levels of IL-17, CD4+ T cells (130) and high expression of MCT (Slc5a12) (129). Hence, lactate is uptake in T cells trough MCT and inhibits glycolysis but increases IL-17 expression through the PKM2/STAT3 pathway. Thus, lactate causes T-cell entrapment and increased expression of cytokines at inflamed sites (131, 132) **(** **Figure 3** **).**

### Lactate in Fibroblast-Like Synoviocytes

Lactate has an important role in inflammatory joint pathologies such as rheumatoid arthritis. Intracellular levels of L-lactate contribute to the production of pro-inflammatory cytokines in FLS through intracellular signaling that involve the MAPK and NF-κB pathways (133). Additionally, it has been proposed that TNF-α-induced IL-6 and IL-8 production in FLS from patients with rheumatoid arthritis (FLS-RA) is dependent on L-lactate levels (133). FLS-RA in a later stage has elevated MCT4 levels (74). The expulsion of lactate by MCT4 protects the cells from the damaging effects of its accumulation. However, it is unknown whether lactate can be taken up and used by other cells. It is highly likely that the role of MCT and metabolite exchange between cells differs during the course of RA depends on the mitochondrial state and the micro-environment of the joint (134).

D-lactate is involved in the etiology of lameness during ARA. In fact, a significant increase in D-lactate has been observed in the synovial fluid of bovines with ARA, and it has been hypothesized that D-lactate may exert a pro-inflammatory effect on bFLS (17). In relation to this, an increase in IL-1β, IL-6, and PGE_2_ has been identified in the synovial fluid of heifers with ARA (18). *In vitro* studies have shown that D-lactate increases IL-6 and IL-8 in bFLS (17, 89). Consistent with this, D-lactate and IL-6 increase early in the synovial fluid of heifers with ARA and this increase occurs before the recruitment of joint neutrophils (19). Similarly, a very early metabolic change with the presence of lactate in arthritic joints precedes the recruitment of phagocytic immune cells (135).

The presence of MCT1 and MCT4 mRNA and proteins has been detected in bFLS. Blocking MCT1 by a selective inhibitor reduces the expression and synthesis of IL-8 and IL-6 (17). These results suggest that lactate plays an important role in inflammatory processes and that entry into cells through MCT could contribute at least in part to exert these effects **(** **Figure 3** **).**

MAPK and NF-κB pathways have been extensively studied and are critical in FLS activation during joint inflammation (136, 137). D-lactate increases ERK1/2 and p38 phosphorylation in bFLS (17), and it has been suggested that L-lactate could also activate p38 and ERK1/2 in FLS-RA (133). Inhibition of these kinases decreased IL-6 and IL-8 induced by D-lactate (17), suggesting that D-lactate could regulate the expression and synthesis of pro-inflammatory cytokines through the MAPK pathway and the NF-κB pathway, which are involved in the synthesis of IL-6, CXCL8 (138) and the expression of COX-2 and mPGES-1 (139, 140) **(** **Figure 3** **)**. D-lactate and bovine TNF-α (bTNF-α) increase the expression and secretion of IL-8 and IL-6 in an NF-κB-dependent manner in bFLS (17). Besides, L-lactate increases the degradation of IκBα and activates NF-κB in human FLS (90, 133). Activation of NF-κB is key for the constitutive secretion of IL-6 and IL-8, as well as the secretion of these cytokines induced by IL-1β in FLS-RA (141), which suggests that lactate in the joint could activate intracellular signaling in FLS leading to the expression of pro-inflammatory markers during joint inflammation.

Overall, the pleiotropic effects of lactate in inflammatory processes could be partially linked by its property as a multifunctional intracellular signaling molecule, which control the transcription factors activity and pro-inflammatory protein expression. Additionally, these effects could be dependent of changes in MCTs expression and lactate metabolism during inflammatory process. In fact, immune cells also show quite dissimilar cellular metabolism, closely related to their role in inflammatory processes, in this scenario lactate differentially affects cell metabolism, and depending on the cell type and metabolic microenvironment, it would exert inflammatory or anti-inflammatory effects. In the past years, the discovery of several G-protein coupled receptors (GPCR) as potential lactate and proton sensors, could additionally explain the varied responses seen with lactate in inflammation (111, 142).

## Lactate as a G Protein-Coupled Receptor Agonist

Hydroxycarboxylic acid receptor 2 (HCA2) is a GPCR also known as PUMA-G (upregulated protein in macrophages by IFN-γ) (143), HM74A, and GPR109A (144). White and brown adipose tissue, macrophages, neutrophils, Langerhans epidermal cells, DCs, and microglia express HCA2 (143, 145–150). Proinflammatory stimuli such as LPS, IL-6, and IL-1β (144) increase the expression of HCA2 in macrophages, and colony-stimulating factor 2 (CSF2) augment the level of HCA2 expression in neutrophils (151). In macrophages, HCA2 couples to Gαi/o-type G proteins that, *via* protein kinase A (PKA) or Gβγ, can inhibit NF-κB and reduces cytokine expression (152), whereas in neutrophils HCA2 *via* Gαi/o reduces cAMP favoring apoptosis through the pro-apoptotic protein BAD (147). In sepsis, lactate-induced activation of HCA2 decreases the inflammatory response by reducing cytokine expression and promoting M2-like polarization (153) **(** **Table 1** **)**.

**Table 1:** Main characteristics of lactate-activated and proton sensor receptors in inflammatory response.

Receptor	Ligand	Location	G protein	Biological Function
**HCA2, PUMA-G, HM74A or GPR109A.**	Lactate	White and brown adipose tissue, macrophages, neutrophils, Langerhans epidermal cells, dendritic cells, and microglia.	Gαi/Gαo	Inhibition of NF-κB and reduction cytokine expression and apoptosis through BAD. Promotion to M2-like polarization of macrophages.
**HCA1 or GPR81**	Lactate	Adipocytes, macrophages monocytes, endothelial cells	Gαi	Inhibition of lipolysis in adipocytes. Inhibition of NLRP3 inflammasome release by activating the intracellular adaptor protein, β-arrestin 2 (ARRB2), and attenuating NF-κB activity. Increased vascular permeability inducing the mobilization of bone marrow (BM) neutrophils.
**GPR4**	Proton sensor	Vascular endothelial cells.	Gαs	Induces NF-κB activation and increases the inflammatory response in endothelial cells. Activates apoptotic pathways and regulates the endoplasmic reticulum (ER) stress in endothelial cells.
**GPR65 or TDAG8**	Proton sensor	T cells, B cells, neutrophils, and eosinophils.	Gαs	Reduces pro-inflammatory cytokine production (TNF-α and IL-6), ROS production and apoptosis.
**GPR132 or G2A**	Proton sensor	Macrophages and neutrophils.	Gαq	Promotes activation of the peroxisome proliferator-activated receptor γ (PPARγ) in tumor-associated macrophages (TAMs) causing activation to macrophage M2 and tumor growth.
**GPR68 or OGR1**	Proton sensor	Macrophages, dendritic cells, T cells and neutrophils.	Gαq/11 and Gαs	Maintain tumor-associated macrophages in an M2-like state and suppresses T-cell infiltration favoring tumor growth. Increases the production of CXCL8 and IL-6 in human airway smooth muscle cells related to bronchial contraction and hyperresponsiveness of the airways in patients with bronchial asthma.

Lactate signaling can be modulated by the G-protein coupled receptor GPR81 (also named HCA1) localized in the cytoplasmic membrane. GPR81 is coupled to Gαi-type G proteins (154). After lactate stimulation, the distribution of GPR81 is observed in intracellular granules, suggesting internalization (155). GPR81 activation occurs at a lactate concentration of 0.2 to 1.0 mM (156), followed by down-regulation of cAMP and inhibition of PKA-mediated signaling (157). Lactate binds to GPR81 in adipocytes inhibiting the lipolysis (158). Evidence suggests that GPR81 is an anti-inflammatory pathway that inhibits NLRP3 inflammasome release by activating the intracellular adaptor protein, β-arrestin 2 (ARRB2), and attenuating NF-κB activity **(** **Figure 3** **)** (159). In macrophages and monocytes, lactate binds to GPR81 and reduces the effects induced by TLR4 agonists such as NF-κB activation, the release of IL-1β, and cleavage of CASP1, *via* ARRB2 (115) **(** **Table 1** **)**. Lactate through GPR81 reduces inflammation and organ injury in mice with auto-immune hepatitis (115). In GPR81-/- mice, susceptibility to dextran sulfate sodium (DSS)-induced colonic inflammation is increased, while pharmacological activation of GPR81 decreases the expression of inflammatory cytokines and improves colonic inflammation (160). GPR81 has also been involved in the anti-inflammatory activity of lactate in mouse uterine inflammation during labor (161). It has recently been demonstrated in macrophages that lactate *via* GPR81/ARRB2 increases acetylation of HMGB1 by inducing nuclear translocation of acetylase p300/CBP resulting in increased endothelium permeability (162).

LPS activates bone marrow (BM) neutrophils and induces lactate release through increased glycolysis. Lactate released acts on GPR81 expressed by endothelial cells to increase vascular permeability inducing the mobilization of BM neutrophils (163). Lactate increases the levels of neutrophil-attracting chemokines favoring rapid neutrophil mobilization from the BM (163). LPS decrease the expression of GPR81 and MCT-1 in endothelial cells and increase lactate concentrations in the extracellular space, suggesting a role in neuroinflammatory processes altering structural integrity of the blood-brain barrier *in vitro* (164). Additional evidence suggests that the activation of GPR81 reduces oxidative stress and the expression of IL-6, IL-8, monocyte chemoattractant protein (MCP)-1 and HMGB1 (165). Accordingly, it has been shown that the activation of GPR81 can exert atheroprotective effects in endothelial cells exposed to oscillatory shear stress (OSS).

The activation of GPR81 inhibits the secretion of the vascular cellular adhesion molecule (VCAM)-1 and endothelial selectin (E-selectin), which suppress monocyte attachment to the endothelium (165). Conversely, lactate induces the expression of the neutrophil chemokines CXCL1, CXCL2, and G-CSF in bone marrow and plasma through a mechanism independent of the GPR81 receptor (163). This suggests a potential pleiotropic pro-inflammatory effect in of lactate; however, it remains to be clarified whether lactate produces its effects by direct activation of the receptor or through lactic acidosis, which could produce conformational modifications in the receptor.

Acidosis is a hallmark of the microenvironment of inflammatory pathologies (166, 167), where lactic acid is among the most important extracellular metabolites (168). Thus, other putative lactate sensors GPR4, GPR65 (TDAG8), GPR68 (also known as ovarian cancer G protein-coupled receptor 1, OGR1), and GPR132 (G2A) have been described as proton sensitive and could be involved in immune modulation during the inflammatory processes characterized by low pH levels obtained from lactic and carbonic acids (166, 169).

GPR132 is described in neutrophils (170) and macrophages and appears to be responsible for migration to recruit macrophages in the pro-inflammatory microenvironment surrounding the focus of inflammation (171). In addition to being the least sensitive to extracellular acidification, it can be stimulated by lysophospholipids (172), generating IP3 through Gαq activation (173). GPR132 expression is reduced by the activation of peroxisome proliferator-activated receptor γ (PPARγ) in tumor-associated macrophages (TAMs); moreover, breast tumor cells produce lactate which activates GPR132 in TAMs, promoting macrophage M2 activation and tumor growth (174, 175).

GPR4 is a pro-inflammatory GPCR that activates the Gs-cAMP-exchange protein activated by cAMP highly expressed in vascular endothelial cells linked to leukocyte adhesion (176). Activation of GPR4 by extracellular acidification also increase the expression of chemokines, cytokines, and adhesion molecules through the NF-κB pathway in endothelial cells **(** **Table 1** **)** (176–178). Acidosis-induced activation of GPR4 promotes the endoplasmic reticulum (ER) stress response and apoptosis of endothelial cells (177, 179, 180). GPR4^-/-^ reduces inflammation in the DSS-induced acute colitis and in the spontaneous IL-10-/- colitis model in rodents (169, 181).

GPR65 (TDAG8) is expressed in T cells, B cells, neutrophils, and eosinophils and is coupled to the Gs/adenyl cyclase/cAMP pathway (182, 183). GPR65 activation reduces pro-inflammatory cytokine production (TNF-α and IL-6) in mouse peritoneal macrophages (184, 185), ROS production in human neutrophils (186) and apoptosis in human eosinophils (166) **(** **Table 1** **)**.

GPR68 (OGR1) is a proton-sensing receptor that can detect decreases in extracellular pH during inflammation. It is expressed in macrophages (184), dendritic cells (187), T cells (188), and neutrophils (186) and has a pro-inflammatory function in colitis (189), asthma through activation of dendritic cells (187), and in murine experimental autoimmune encephalomyelitis that regulates T cell responses during autoimmunity (190). This receptor has been described to be able to couple Gαq/11 and Gαs and trigger increased intracellular calcium and cAMP **(** **Table 1** **)** (191, 192). GPR68 may maintain TAM in an M2-like phenotype and inhibits T-cell infiltration, which promotes tumor growth (188). GPR68 expression can be induced by TNF-α in the human macrophage lineage and primary human monocytes, activating Gαq signaling during the development of mucosal inflammation (189, 193). Furthermore, hypoxia improves the TNF-mediated induction of OGR1 expression, which is reversed by NF-κB inhibitors (194). Extracellular acidification induces the production of CXCL8 and IL-6 through OGR1 in human airway smooth muscle cells and could be related to bronchial contraction and hyperresponsiveness of the airways in patients with bronchial asthma (195, 196).

## Conclusions

Lactate, more than a product of metabolism, exerts modulating effects on the immune response and, depending on the cell type, could interfere or promote the inflammatory response. Apparently, the diversity of effects would depend on the pathway by which lactate is generated or metabolized. In addition, during the development of inflammatory processes, various changes occur in cell metabolism, in this scenario the presence of lactate can contribute to enhance or interfere with the immune response, depending on the pathological context analyzed. Furthermore, the evidence suggests that lactate may play as pleiotropic physiological signaling agent, modulating several signal transduction pathways and transcription factors. Lactate can mediate its effects directly through lactate-sensitive G-protein coupled receptors or indirectly through its effects on extracellular acidification, which would stimulate different proton-sensitive receptors. The activation of these receptors can contribute or interfere with the inflammatory process.

Taken together, all the currently available evidence suggest that lactate possesses a myriad of biological effects, which could explain dissimilar responses observed in inflammatory processes.
