# Corrigendum: Role of lactate in inflammatory processes: friend or foe

**DOI:** 10.3389/fimmu.2025.1553925

**Published:** 2025-01-27

**Authors:** Carolina Manosalva, John Quiroga, Alejandra I. Hidalgo, Pablo Alarcón, Nicolás Ansoleaga, María Angélica Hidalgo, Rafael Agustín Burgos

**Affiliations:** ^1^ Faculty of Sciences, Institute of Pharmacy, Universidad Austral de Chile, Valdivia, Chile; ^2^ Laboratory of Immunometabolism, Faculty of Veterinary Sciences, Institute of Pharmacology and Morphophysiology, Universidad Austral de Chile, Valdivia, Chile; ^3^ Graduate School, Faculty of Veterinary Sciences, Universidad Austral de Chile, Valdivia, Chile

**Keywords:** lactate, inflammation, G-protein coupled receptors, immunometabolism, monocarboxylate transport

In the published article, an author name was incorrectly written as “Anseoleaga”. The correct spelling is “Ansoleaga”.

The authors apologize for this error and state that this does not change the scientific conclusions of the article in any way. The original article has been updated.

